# Efficacy and Safety of an Herbal Therapy in Patients with Amnestic Mild Cognitive Impairment: A 24-Week Randomized Phase III Trial

**DOI:** 10.1155/2017/4251747

**Published:** 2017-05-17

**Authors:** Jinzhou Tian, Jing Shi, Tao Li, Lin Li, Zhiliang Wang, Xiaobin Li, Zhu Lv, Qingshan Zheng, Mingqing Wei, Yongyan Wang

**Affiliations:** ^1^Third Department of Neurology, Dongzhimen Hospital, Beijing University of Chinese Medicine, Beijing 100700, China; ^2^Department of Neurology, Xiyuan Hospital, China Academy of Chinese Medical Sciences, Beijing 100091, China; ^3^Laboratory of Pharmacy, Xuanwu Hospital, Capital University of Medical Sciences, Beijing 100053, China; ^4^North China Pharmaceutical Group Corporation, Hebei 050015, China; ^5^Drug Clinical Trial Centre, Shanghai University of Chinese Medicine, Shanghai 200032, China; ^6^Institute of Clinical Medicine, China Academy of Chinese Medical Sciences, Beijing 100700, China

## Abstract

**Objective:**

In the 24-week randomized, double-blind, double-placebo, parallel-controlled trial, we aimed to test the effects of herbal therapy with amnestic mild cognitive impairment (aMCI).

**Methods:**

A total of 324 patients with aMCI entered a 2-week placebo run-in period followed by 24 weeks' treatment of either (a) herbal capsule (5 shenwu capsules/administration, 3 times/day) and placebo identical to donepezil tablets (*n* = 216) or (b) donepezil (5 mg/day) and placebo identical to herbal capsule (*n* = 108).

**Results:**

Herbal therapy showed a significant improvement on the primary efficacy measure, measured by Alzheimer Disease Assessment Scale-cognitive subscale (ADAS-cog), and showed a mean decrease from baseline of 4.23 points at the endpoint, without a significant difference from the donepezil group. Secondary efficacy measurement of the Logical Memory II Delayed Story Recall subtest (DSR) showed modest improvement in those taking herbal capsule compared to baseline, and there was no significant difference from donepezil group. The frequency of adverse events was much less in the herbal therapy group than the donepezil.

**Conclusion:**

Herbal therapy demonstrated a significant improvement in cognition and memory, which were similar to the donepezil in patients with aMCI. Herbal therapy was safe and well tolerated.

**Trial Registration:**

This study is registered with clinicaltrials.gov NCT01451749.

## 1. Background

Mild cognitive impairment (MCI) refers to a group of individuals who have cognitive impairments that are of insufficient severity to constitute dementia [1], which is a transitional stage between normal aging and dementia of Alzheimer's disease (AD) [2]. Amnestic MCI (aMCI) is the most common subtype of MCI syndrome, which is defined as a significant impairment in episodic memory with no impairment in activities of daily living [3]. In an interventional study of patients meeting Petersen's criteria for aMCI, 16% progressed to dementia each year, 99% of whom received an AD diagnosis [4]. Hence, MCI, in particular aMCI, is generally recognized in many cases to represent a prodromal stage of AD [5, 6] and is a treatment target for AD [4–7].

The ultimate aim of AD therapy is to stop or slow down the disease progression. However, the well-studied conventional treatments for AD are generally considered to be symptom-relieving rather than disease-modifying; with that cholinesterase inhibitors (ChEI) treatment for mild to moderate AD may reach peak benefit for cognitive improvement at 3 months but drop below baseline level at 9 months [8, 9]. Patients with moderate to severe AD receiving stable doses of both donepezil and memantine experienced limited cognitive improvement [10]. The use of herbal therapy in the treatment of dementia started from The* Complete Work of Jingyue* published in China in 1624, which contains the earliest known description in the world of an herbal therapeutic strategy for dementia [11, 12]. This herbal capsule (shenwu capsule) is one kind of a lot of herbal therapies that also improved memory and cognitive symptoms in patients with aMCI in a 12-week multicenter, randomized, double-blind phase II trial [13]. The aim of this phase III trial was to further evaluate the effects and safety of the herbal capsule, over 6 months, on cognition and memory in patients with aMCI.

## 2. Subjects and Methods

### 2.1. Participants

**Figure 1:**
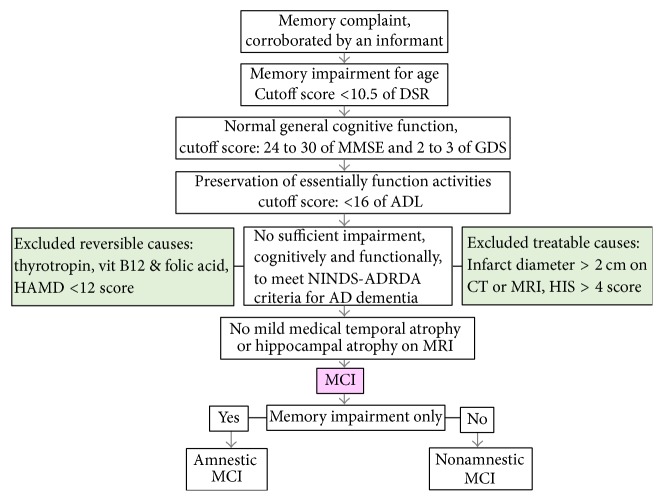
Diagnostic algorithm for aMCI inclusion.* Notes*. ADL = Instrument Activities of Daily Living; HAMD = Hamilton Depression Rating scale; HIS = Hachinski Ischemia scale; MRI = Magnetic Resonance Imaging; NINCDS-ADRDA = National Institute of Neurological and Communicative Disorders and Stroke and the Alzheimer's Disease and Related Disorders Association.

Chinese-speaking adults 55 to 80 years of age, weighing between 45 and 90 kilograms, living in the community were eligible to participate. All patients were recruited from memory clinics in China. The patients were required to meet the diagnostic criteria for MCI as developed by Petersen et al. [4]. The following were the operational criteria (Chinese version) for inclusion in the aMCI group at screening: (1) memory complaints that were corroborated by an informant, (2) abnormal memory function as assessed by Chinese version of the Adult Memory and Information Processing Battery (AMIPB) Logical Memory Delayed Story Recall (DSR) subtest score <10.5 for age (cutoff scores: ≤11.15 for 50 to 60 years old, ≤10.55 for 61 to 75 years old, ≤8.1 for 76 to 85 years old.) [14], (3) normal general cognitive function as determined by a clinician's judgment based on a structured interview with the patients (a Mini-Mental State Examination: MMSE of 24 to 30 score for education) [15], (4) no or minimal impairments in activities of daily living as determined by a clinical interview with the patient and an informant (an Activities of Daily Living: ADL < 16 score) [16], and (5) being not sufficiently impaired, cognitively and functionally, to meet the National Institute of Neurological and Communicative Disorders and Stroke and Alzheimer's Disease and Related Disorders Association criteria for AD [17] as judged by an experienced AD research clinician. In addition, they were judged to be at stage 2-3 of the Global Deterioration scale (GDS) [18], to have a score of ≤12 of the Hamilton Depression Rating scale (HAMD for 17 items) [19] and ≤4 on the Hachinski Ischemia scale (HIS) [20], and to have no or mild medial temporal atrophy (MTA) or hippocampal volume atrophy on a magnetic resonance imaging (MRI) scan [21]. These operational Petersen criteria for inclusion in the aMCI group were modified from the criteria used in one of our previous studies (Figure 1) [22] and are largely consistent with those used in previous donepezil MCI studies [4, 7]. The subjects were required to have adequate vision and hearing to participate in the study assessments.

Patients were excluded if they met any of the following criteria: an MMSE score of ≤19 for illiteracy, ≤22 for primary school, or ≤26 for middle school, evidence of a focal brain lesion, a previous head injury with loss of consciousness or immediate confusion after the injury, a history of significant cerebrovascular disease, a central nervous system infarct or infection, focal lesions of clinical significance on a computerized tomography scan or MRI, any history of a major psychiatric disorder including DSM-IV-defined psychosis, major depression, bipolar disorder, or alcohol or substance abuse [23], documented evidence of an active gastric or duodenal ulcer within the previous 3 months, a history of active malignancy or prostate cancer within the preceding 24 months, a chronic or acute renal, hepatic, or metabolic disorder, uncontrolled hypertension or diabetes mellitus, a neurologic disease that might affect cognition such as AD, Parkinson's disease, Huntington disease, multiple sclerosis, normal pressure hydrocephalus, epilepsy, cerebral tumor, and toxic metabolic encephalopathy, a history of hypersensitivity to clinical drugs including anticonvulsant, antiparkinsonian agents, antipsychotics, anxiolytics, hypnotics, neuroleptics, cholinomimetics, vitamin E, ginkgo biloba extract, or any other drugs that can affect memory, or participating in any other clinical studies within the past 30 days.

### 2.2. Study Design

Subjects enrolled in this clinical trial were assigned at a ratio of 2 : 1 to the herbal group and the donepezil group. The sample size was determined based on a previous report [24]. According to that report, the improvements in the patients who received donepezil treatment at 5–10 mg/day was 3.3 (±4.7) points on Alzheimer's disease Assessment Scale-cognitive subscale (ADAS-cog). The sample sizes of 168 for the herbal group and 84 for the donepezil group were estimated based on changes in ADAS-cog scores of 1.5 points, with a power of 80% at a 0.025 significance level (single side). Because of the greater rate of discontinuation (22%) in this study, the sample size was increased to 216 for the herbal group and 108 for the donepezil group.

In this multicenter, randomized, double-blind, double-placebo, parallel-controlled phase III trial, total 324 were randomly assigned to the herbal group or the donepezil group. The patients entered a 2-week placebo run-in period, and all patients received a MRI scan before being randomized and then were randomized (2 : 1) to receive 24 weeks of treatment with either (a) herbal capsule (5 capsules/time, 3 times/day) and a placebo that was identical to donepezil tablets (*n* = 216) or (b) donepezil (5 mg/day) and a placebo that was identical to herbal capsules (*n* = 108). Active drug was supplied in an herbal capsule (shenwu capsule) containing a 451 mg extract from* Panax ginseng* (4.24%), radix polygoni multiflori (21.28%),* Epimedium brevicornum* Maxim (14.89%),* Acorus tatarinowii* Schott (14.89%), chuanxiong (14.90%), and lobed kudzuvine root (29.80%). Another active drug was a donepezil 5 mg tablet. The placebo medications were identical to the active medications. The herbal capsules were supplied by the sponsor, the Northern China Pharmaceutical Group Corporation (Hebei province, China). The donepezil hydrochloride (Aricept) was supplied by Eisai, Inc.

Randomization was stratified according to the study sites using the SAS statistical software (version 6.12). The randomized code was generated by a central randomization schema by statistics. The subjects, investigators, and sponsor were blinded to treatment allocation.

We followed up with all patients who were enrolled. Study visits take place at screening (day 1 clinic visit), at mid-study (week 12), and at the endpoint of treatment (week 24). Patients who completed the 24 weeks of the study were followed up 24 weeks after withdrawal.

This study was undertaken in accordance with the principles of the Declaration of Helsinki and the International Conference on Harmonization of Technical Requirements for Registration of Pharmaceuticals for Human Use guidelines for good clinical practice. The study protocol was approved by the medical ethics boards of the study institutions where the principal investigators worked. Nine institutions of national drug clinical trials participated in this study. All patients and their caregivers provided written informed consent.

All treatments were documented, including the name of drug, the daily dose, the reason for its use, and the date of termination.

### 2.3. Efficacy Measurements

The primary efficacy outcomes measured cognition. The secondary efficacy outcomes measured memory. Cognition was assessed with the ADAS-cog [25], and memory was evaluated with the DSR [14].

The ADAS-cog was designed specifically to evaluate the severity of cognitive dysfunctions characteristic of AD patients and includes 11 items. Among these items, memory, orientation, language function, practical ability, and attention are evaluated. The score on the ADAS-cog ranges from 0 to 70 points, with 0 points indicating no impairment and 70 points indicating severe impairment of cognition.

The secondary efficacy measures were the logical memory subtest, which was used to evaluate the memory of a patient and is often used not only as a screening tool, but also as a secondary efficacy measure in clinical trials of MCI [7]. Inclusion of the delayed recall condition of a story memory task enhanced the overall accuracy of distinguishing MCI from normal aging (sensitivity = 92.2%; specificity = 94.7%), and this clinical measure was found to have a positive predictive value (PPV) of around 85% [26]. The DSR Chinese version from the AMIPB also has higher sensitivity (90%) and specificity (80%) for screening MCI or very mild AD (CDR = 0.5) [14, 22].

### 2.4. Safety Assessments

Information on adverse events and compliance in the donepezil and herbal therapy groups was collected during all of the postbaseline study visits (week 1 and every 3 months until the end of the study) and via additional telephone conversations with caregivers at week 2 and every month until the end of the study. The safety assessment included (1) examinations of general physical vital signs including breathing, heart rate, and blood pressure, (2) electrocardiography (ECG), (3) laboratory testing, and (4) documentation of any adverse events that occurred including the type of event, when it occurred, its duration, treatment measures, the likely relationship between the tested drugs and the adverse events (positive, probable, possible, and not related), and the severity of the event (mild, moderate, and severe).

### 2.5. Statistical Analysis and Power Calculations

The statistical analyses were conducted using SAS software. In the randomized trial, the analyses for efficacy were conducted in two patient populations: the intent-to-treat population (ITT) and the fully evaluable population (FE). The ITT population includes every subject who is randomized. Primary and secondary efficacy analyses were conducted using last observation carried forward (LOCF) methods for missing data. The FE population included patients who completed 24 weeks of the medication with good compliance and with complete data. The safety sample included all subjects who received at least one dosing and at least one safety evaluation. The therapeutic window for efficacy evaluation was extended to include 7 days after the last dose of a study drug.

The changes from baseline on the ADAS-cog and DSR were determined as an efficacy measurement by application of covariance analysis that controlled for the baseline score and center effect. We compared the demographic variables between the two groups using a *t*-test for age and weight and Fisher's test for education and race. The reported prevalence of adverse events between the two groups was compared by Pearson's chi-square test. All *p* values were two-tailed, and all analyses were significant if the *p* value was ≤0.05. The analyses of safety were conducted in the safety population.

This phase III study is registered with ClinicalTrials.gov (Identifier: NCT01451749).

## 3. Results

**Figure 2:**
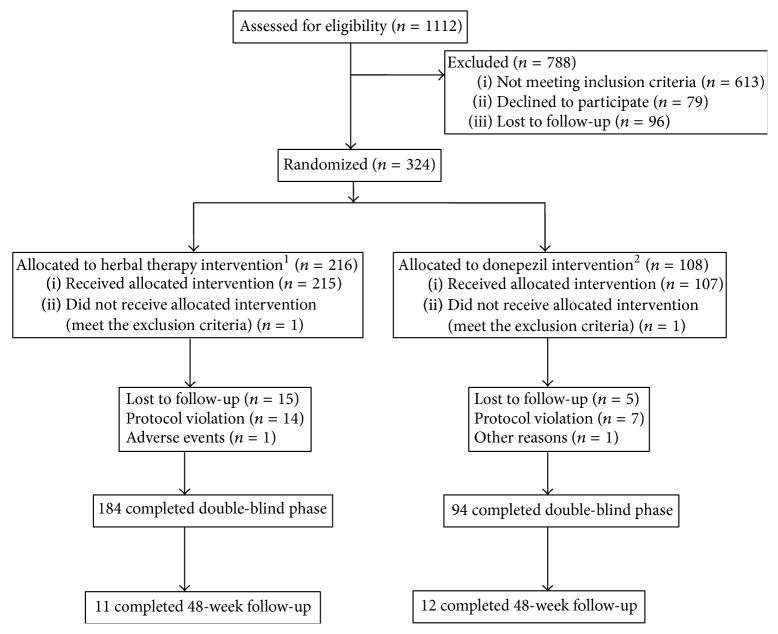
Study design, subject allocation, and subject course.* Notes*. ^1^The herbal group means the patients who took the herbal capsule and placebo donepezil tablets. ^2^The donepezil group means the patients who took donepezil and placebo herbal capsules.

Nine study sites in China were used to examine the patients from September 1, 2008, to May 3, 2010. Of the 1112 subjects screened in these nine sites (Figure 2), 613 subjects were excluded because they did not meet the inclusion criteria, withdrew consent (79 subjects), or were lost to follow-up (96 subjects). A total of 324 eligible subjects were randomized into the herbal capsule (*n* = 216) or the donepezil tablet group (*n* = 108) and received at least one dose of the study medication. One subject in the herbal therapy group and one subject in the donepezil group did not meet the inclusion criteria, 215 subjects were allocated to the herbal therapy group, and 107 subjects were allocated to the donepezil group. Of these 322 patients, 44 discontinued their treatment before week 24. The remaining 184 subjects in the herbal therapy group and 94 in the donepezil group completed the study. Premature discontinuations were because the subject did not complete the follow-up (14 patients in the herbal therapy group and 5 patients in the donepezil group), adverse events occurred (2 patients in the herbal therapy group), or a protocol violation occurred (14 patients in the herbal therapy group and 7 patients in the donepezil group). In two cases, the subjects refused to explain the reason for their withdrawal (one in each group).

The demographic characteristics of the subjects at baseline are summarized in Table 1. Baseline demographic characteristics were similar in the two groups. There were no significant differences between the two groups with respect to age, sex, race, education, or baseline neuropsychological and cognitive test scores (e.g., MMSE, GDS, and HAMD). At the end of the study, there was no significant difference in compliance between the herbal therapy (92.6%) and the donepezil group (95.3%) (Fisher's test, *p* = 0.474).

### 3.1. Efficacy Measurement

#### 3.1.1. Primary Efficacy Measurement

**Figure 3:**
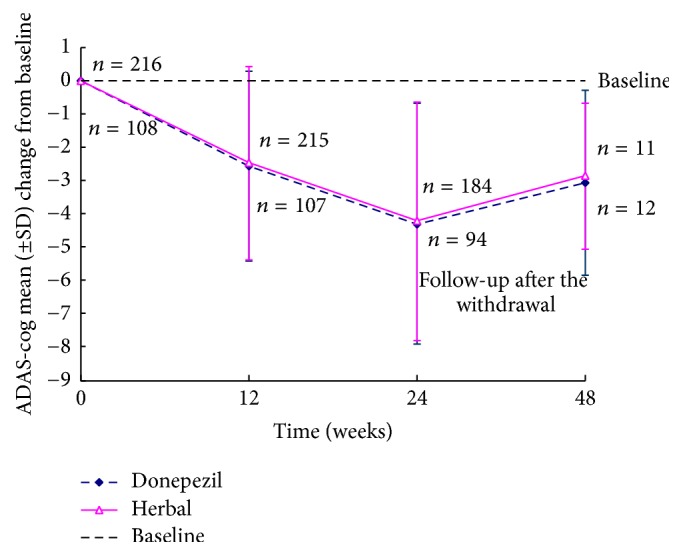
ADAS-cog outcome: mean change from baseline in patients with aMCI (ITT-LOCF analysis).* Notes*. ADAS-cog = Alzheimer's Disease Assessment Scale-cognitive subscale. Graphs show data at baseline and each assessment point.

There were significant differences in the mean change from baseline of ADAS-cog between the two groups (*p* < 0.001). Mean baseline ADAS-cog scores were 14.83 (6.39) in the herbal therapy group and 15.14 (6.10) in the donepezil group and 14.72 (6.50) (Table 1). At the study endpoint, compared with baseline, significant improvements on ADAS-cog scores were observed in the two treatment groups (ITT-LOCF and FE analyses) (Table 2), and these improvement were not significantly different between the 9 different centers (*p* = 0.847). In the herbal therapy group, mean changes (SD) from baseline of the ADAS-cog were −4.23 (3.57) [95% CI −4.71 to −3.75], and they were −4.31 (3.61) [95% CI −4.99 to −3.63] in the donepezil group (ITT-LOCF analysis, Figure 3), both of which represent significant improvements compared with baseline (all *p* < 0.001). No significant difference was observed in the change of the ADAS-cog scores from baseline to the study endpoint between the two groups (Student's *t*-test, *p* = 0.851). Because the 97.5% CI of the treatment difference between the herbal therapy group and the donepezil group (−0.914 to 0.754) was greater than the noninferiority interval (−1.5~1.5), herbal therapy was not inferior to donepezil.

**Figure 4:**
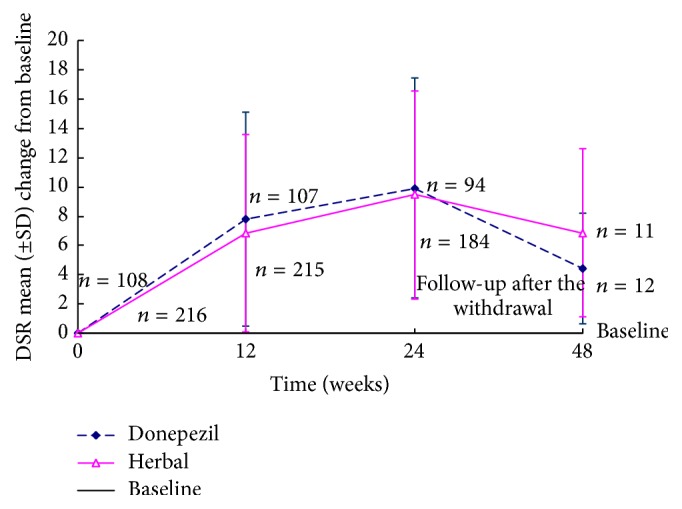
Secondary outcome: mean change of DSR scores from baseline in patients with aMCI (ITT-LOCF analysis).* Notes*. DSR = Delayed Story Recall.

The differences between groups in the mean change from baseline to the study endpoint based on the FE analysis [herbal therapy: −4.58 (3.55), donepezil: −4.53 (3.69); *p* = 0.915] were significant (all *p* < 0.001). Because the 97.5% CI of the treatment difference between the two groups (−0.848 to 0.948) was greater than noninferiority interval (−1.5~1.5), herbal therapy was not inferior to donepezil. This difference was not present at the follow-up conducted 24 weeks after drug discontinuation for either group (Figure 4).

#### 3.1.2. Secondary Efficacy Measurement

There were significant differences between the two groups with regard to the mean changes in the DSR (*p* < 0.001) scores. Changes in the DSR scores at the study endpoint were similar for the herbal therapy and donepezil groups in the LOCF analysis of the ITT population, the mean increase of the DSR scores being 9.45 (7.08) [95% CI 8.50 to 10.40] in the herbal therapy group and 9.92 (7.53) [95% CI 8.49 to 11.34] in the donepezil group. Both showed significant improvements compared to baseline (all *p* < 0.001), but there was no significant difference between the two groups (*p* = 0.587).

At the study endpoint, analyses of the FE populations showed significant improvement in both the herbal therapy group, 10.07 (6.73), and the donepezil group, 10.30 (7.58), compared to baseline (all *p* < 0.001), but there were no significant differences between the two groups (*p* = 0.794) (Table 2). This difference was not present at the follow-up conducted 24 weeks after drug discontinuation for either group (Figure 4).

#### 3.1.3. Conversion Outcomes

Conversion rates of MCI to AD were analyzed based on the small number of subjects at only the Dongzhimen Hospital site at which 11 of the patients in the ITT population in the herbal therapy group and 12 of the patients in the ITT population in the donepezil group were followed up at week 24 after the study endpoint. None of the patients with aMCI converted to AD in the two treatment groups, and there were no significant differences between the three groups (*p* = 0.408). There was also no significant difference in recovery to normal cognition between the two groups (*p* = 0.167 for 18.2% in the herbal therapy group, 16.7% in the donepezil group). Most patients with aMCI remained stable 24 weeks after the study endpoint, and there were no differences between the two treatment groups (*p* = 0.398 for 81.8% in the herbal therapy group, 83.3% in the donepezil) (Table 3). It should be noted that data were available only from a very small sample, and the results may not be representative of the overall outcome.

### 3.2. Safety and Tolerability

During the study, 18.5% (39/216) of patients reported adverse event in the herbal therapy group and 57.4% (62/108) of patients reported adverse events in the donepezil group (Table 4). The prevalence of probable and possible adverse events in the herbal therapy group was similar to that in the donepezil group (Chi-square test, *p* = 0.259). The most frequent adverse events assessed as probably related to the study medication in the herbal therapy group were thirst (6.5%) and sore throats (4.2%). The prevalence of diarrhea (0%), nausea (2.1%), and insomnia or abnormal dreams (2.3%) in the herbal therapy group was significantly lower than the rates of 12.5% (*p* < 0.05), 16.7% (*p* < 0.05), and 16.7% (*p* < 0.05), respectively, in the donepezil group. Two patients who received herbal therapy discontinued the study because of adverse events or intolerance. All of the adverse events were mild to moderate (Table 4), and there were no severe adverse events in either of the two treatment groups. No significant changes from baseline were observed in vital signs, physical examination findings, ECG status, or laboratory values between the two treatment groups.

## 4. Discussion

At present, there are no approved treatments for MCI. Studies have shown that donepezil significantly improves ADAS-cog scores, which measures cognition [7]. Herbal therapy capsule is the first traditional Chinese herbal medicine evaluated in approved clinical trials for the treatment of MCI and the mild to moderate dementia in AD by the State Administration of Food and Drugs of China. The aim of this 24-week, donepezil-controlled phase III trial was to further evaluate the effectiveness of herbal therapy as a traditional Chinese herbal monotherapy for patients with aMCI.

MCI represents an intermediary stage between normal cognition and mild dementia. At present, the diagnosis of MCI relies on objective measures such as MMSE and LMS scores. In this study, we used the Chinese version of these operational criteria for inclusion in the aMCI group. The mean MMSE score in this group was 27 points, which is similar to findings in previous studies [7, 27]. This indicates that the Chinese version of the operational criteria for aMCI is reliable.

In this study, treatment with 15 capsules of herbal therapy per day produced a significant improvement in ADAS-cog scores, with a decrease of 4.19 points from baseline in patients with aMCI, which was not significantly different than the decrease in the donepezil group. According to previous studies, an improvement of 3.3 points or more in ADAS-cog scores with antidementia therapy would be considered a clinically significant effect [28, 29]. This study shows that herbal therapy has similar clinical benefits on MCI to donepezil.

Episodic memory is the first and most severely affected cognitive domain and is also a core feature of the diagnosis of AD or aMCI [6, 7]. The herbal-treated group showed significant improvements on secondary DSR measures of memory compared to baseline (*p* < 0.001) that were compared to those seen in the donepezil group.

The herbal therapy and donepezil were safe and well tolerated. The frequency of adverse events differed greatly between the herbal therapy and donepezil groups. 57.4% of the subjects reported at least one adverse event in the group that received donepezil at 5 mg/day for 24 weeks. In contrast, only 18.0% of subjects reported experiencing at least one adverse event in the herbal therapy group. The most frequent adverse events assessed as probably related to the study medication in the herbal therapy group were thirst, sore throat, insomnia or abnormal dreams, and nausea. These occurred at significantly lower rates than in the donepezil group.

There are some limitations of this study. First, there was no placebo group. As all subjects knew that they were being treated with one of two drugs, and all of the individuals who assessed the patients knew this as well, our results may have been influenced by a positive response bias. Second, the sample size was relatively small. Third, period of follow-up was relatively short. Hence, further studies with a placebo group, a large scale, and a long-term follow-up must be conducted to fully evaluate the efficacy of herbal therapy in patients with MCI.

## 5. Conclusion

In summary, this phase III study provides evidence of a possible therapeutic effect of herbal therapy in patients with aMCI. Compared to baseline, herbal therapy produced significant improvements in ADAS-cog measures of cognition and DSR measures of memory in both the ITT population and FE population. Moreover, the herbal therapy was generally safe and well tolerated in this study up to 24 weeks of treatment. We suggest that herbal therapy may be an effective therapy for the patients with MCI. Further studies should be conducted using long-term therapeutic interventions to investigate whether the herbal therapy delays the progression from MCI to dementia.

## Figures and Tables

**Table 1:** Baseline demographic and clinical characteristics in the patients with aMCI (ITT population).

	Herbal therapy	Donepezil	^*∗*^ *p*
	*n* = 215	*n* = 107	
Age (year)	62.67 (7.96)	63.80 (8.25)	0.332
Gender			
Male	88 (40.9)	46 (43.0)	0.724
Female	127 (59.1)	61 (57.0)	
Education			
Primary school	40 (18.6)	26 (24.3)	0.233
Longer than middle school	175 (82.4)	81 (75.7)	
Race *n* (%)			
Han	207 (96.3)	106 (99.1)	0.281
Others	8 (3.7)	1 (0.9)	
MMSE score	27.38 (1.35)	27.11 (1.59)	0.140
HAMD score	6.44 (3.27)	6.39 (3.51)	0.910
GDS stage			
Stage 1	0 (0)	1 (0.9)	0.843
Stage 2	101 (47.0)	47 (43.9)	
Stage 3	114 (53.0)	59 (55.1)	
ADAS-cog	14.81 (6.39)	15.14(6.10)	0.650
DSR	10.03 (2.97)	9.90 (3.25)	0.719

Data are mean (SD) or number (%). ^*∗*^*p* value for the comparison between the donepezil group and herbal group and the nontreated group. ITT = intent-to-treat. ADAS-cog = Alzheimer's Disease Assessment Scale-cognitive subscale; DSR = Delayed Story Recall; HAMD = Hamilton Depression Rating scale; HIS = Hachinski Ischemia scale; GDS = Global Deterioration Scale; MMSE = Mini-Mental State Examination.

**Table 2:** Changes from baseline to the end of the study on efficacy measures after 24 weeks of treatment in patients with aMCI (ITT-LOCF analyses).

Change from baseline to endpoint	Herbal therapy		Donepezil		^*∗*^ *p* value
	*n* = 215		*n* = 107		
	Mean (SD)	95% CI	Mean (SD)	95% CI	
ADAS-cog	−4.23 (3.57)	−4.71~−3.75	−4.31 (3.61)	−4.99~−3.63	0.000
DSR	+9.45 (7.08)	+8.50~10.40	+9.9 (7.53)	+8.49~11.34	0.000

Data are mean (SD) changes in score from baseline to 24 weeks.^*∗*^*p* value for the comparison between the donepezil group and herbal group of the mean change from baseline. ADAS-cog = Alzheimer's Disease Assessment Scale-cognitive subscale; DSR = Delayed Story Recall.

**Table 3:** Comparison of conversion outcomes after one year between the different groups.

	Herbal therapy	Donepezil	^*∗*^ *p* value
	*n* = 11	*n* = 12	
Progressed to AD	0 (0%)	0 (0%)	0.408
Converted to NC	2 (18.2%)	2 (16.7%)	0.167
Stable MCI	9 (81.8%)	10 (83.3%)	0.398

Data are number (%) of patients. *∗* indicates *p* value for the comparison between herbal group and donepezil group.

**Table 4:** Adverse events in the safety population.

Adverse events^§^	Herbal therapy		Donepezil	
	(*n* = 216)		(*n* = 108)	
Any adverse events *n*	39	18.0%	62	57.4%
Abnormal alanine aminotransferase (ALT)	1	0.5%	1	0.9%
Insomnia or abnormal dreams	5^*∗*^	2.3%^*∗*^	18	16.7%
Skin rash	0	0%	1	0.9%
Nausea	5^*∗*^	2.1%^*∗*^	18	16.7%
Vomit	0	0%	5	4.2%
Diarrhea	0^*∗*^	0%^*∗*^	14	12.5%
Thirsty	14	6.5%	5	4.2%
Sore throat	9	4.2%	0	0%
Upper respiratory tract infection	5	2.3%	0	0%

Value are *n* (%) of subjects. ^§^Trial drug relationships considered probably and possibly drug related. *∗* indicates *p* < 0.05 for the comparison with donepezil group.

## Abbreviations

AChE: Acetylcholinesterase
AD: Alzheimer's disease
ADAS-cog: Alzheimer's Disease Assessment Scale-cognitive subscale
APP: Amyloid precursor protein
GDS: Global Deterioration scale
ChAT: Choline acetyl transferase
DSR: Delayed story recall
HIS: Hachinski Ischemia scale
FE: Fully evaluable
HAMD: Hamilton Depression Rating scale
ADL: Activities of Daily Living
ITT: Intent-to-treat
LOCF: Last observation carried forward
MMSE: Mini-Mental State Examination
MCI: Mild cognitive impairment
NINCDS-ADRDA: National Institute of Neurological and Communicative Disorders and Stroke and Alzheimer's Disease and Related Disorders Association
DSM-IV: Diagnostic and Statistical Manual of Mental Disorders, fourth edition.
